# PREVALENCE OF METABOLIC COMORBIDITIES AMONG COGNITIVELY NORMAL AND IMPAIRED WHITE AND AFRICAN AMERICANS

**DOI:** 10.1093/geroni/igad104.3535

**Published:** 2023-12-21

**Authors:** Mohammad Turaani, Subhamoy Pal, Jonathan Reader, Bruno Giordani, Voyko Kavcic

**Affiliations:** Wayne State University, Detroit, Michigan, United States; Michigan Alzheimer’s Disease Research Center, Ann Arbor, Michigan, United States; University of Michigan, Ann Arbor, Michigan, United States; University of Michigan, Ann Arbor, Michigan, United States; Wayne State University, Detroit, Michigan, United States

## Abstract

Assessing comorbidities associated with a MCI diagnosis is crucial for diagnostic accuracy and for understanding the role of comorbidities in cognitive decline. In this study of amnestic (aMCI) and non-amnestic (naMCI) MCI participants and persons with normal cognition (CN), we compared the prevalence of three primary comorbidities: Hypertension (HTN), Hyperlipidemia (HLD), and Diabetes Mellitus (DM) across African American and White populations using Chi-squares. Participant data (N = 9,342, 17% African American) were available through the National Alzheimer’s Coordinating Center and included: CN (n = 5,963; MOCA mean=27), aMCI (2,694; MOCA=22), and naMCI (685; MOCA=24) with diagnosis and data per their first Uniform Data Set (Version 3) visit. Significant differences in the distribution of HTN, HLD, and DM were found among the diagnostic groups for the total cohort and racial groups, separately; however, diagnostic differences across races were not always consistent. The relative rates of DM and HLD across the diagnostic groups for both races were generally similar, though higher percentages were seen in African Americans (25% of African Americans, 10% of Whites). As for HTN (52% of African Americans, 39% of Whites), however, the distributions differ across the diagnostic groups and race (%yes for diagnosis; White Americans: CN 35%, aMCI 47%, naMCI 44%; African Americans: CN 66%, aMCI 70%, naMCI 79%, p< 0.001). These findings highlight the importance of considering the contributions of both race and diagnosis when evaluating the role of comorbid factors and metabolic disorders in NC and MCI groups, in particular when considering blood pressure-related measures.

